# Eastern equine encephalitis virus in mice II: pathogenesis is dependent on route of exposure

**DOI:** 10.1186/s12985-015-0385-2

**Published:** 2015-09-30

**Authors:** Shelley P. Honnold, Eric C. Mossel, Russell R. Bakken, Cathleen M. Lind, Jeffrey W. Cohen, Lori T. Eccleston, Kevin B. Spurgers, Rebecca Erwin-Cohen, Pamela J. Glass, Radha K. Maheshwari

**Affiliations:** Virology Division, United States Army Medical Research Institute of Infectious Diseases, Fort Detrick, Frederick, MD 21702 USA; Department of Pathology, Uniformed Services University of the Health Sciences, 4301 Jones Bridge Road, Bethesda, MD 20814 USA

**Keywords:** Alphavirus, Eastern equine encephalitis virus, Neuroinvasion, Mice

## Abstract

**Background:**

Eastern equine encephalitis virus (EEEV) is an alphavirus with a case fatality rate estimated to be as high as 75 % in humans and 90 % in horses. Surviving patients often have long-lasting and severe neurological sequelae. At present, there is no licensed vaccine or therapeutic for EEEV infection. This study completes the clinical and pathological analysis of mice infected with a North American strain of EEEV by three different routes: aerosol, intranasal, and subcutaneous. Such an understanding is imperative for use of the mouse model in vaccine and antiviral drug development.

**Methods:**

Twelve-week-old female BALB/c mice were infected with EEEV strain FL93-939 by the intranasal, aerosol, or subcutaneous route. Mice were euthanized 6 hpi through 8 dpi and tissues were harvested for histopathological and immunohistochemical analysis.

**Results:**

Viral antigen was detected in the olfactory bulb as early as 1–2 dpi in aerosol and intranasal infected mice. However, histologic lesions in the brain were evident about 24 hours earlier (3 dpi vs 4 dpi), and were more pronounced following aerosol infection relative to intranasal infection. Following subcutaneous infection, viral antigen was also detected in the olfactory bulb, though not as routinely or as early. Significant histologic lesions were not observed until 6 dpi.

**Conclusion:**

These pathologic studies suggest EEEV enters the brain through the olfactory system when mice are exposed via the intranasal and aerosol routes. In contrast, the histopathologic lesions were delayed in the subcutaneous group and it appears the virus may utilize both the vascular and olfactory routes to enter the brain when mice are exposed to EEEV subcutaneously.

## Background

Eastern equine encephalitis viruses (EEEV), genus *Alphavirus* in the family *Togaviridae*, are single-strand, positive-sense RNA viruses that causes significant morbidity and mortality in infected horses, birds, and humans [1]. There are four antigenic subtypes of EEEV, one that circulates in North America and the Caribbean (NA EEEV), and three that circulate in Central and South America (formerly SA EEEV, recently redesignated Madariaga virus (MADV)) [2]. The strains differ in their geographic, epidemiologic, pathogenic, phylogenetic, and evolutionary characteristics. NA EEEV strains are highly conserved, monophyletic, and temporally related, while MADV strains are highly divergent, polyphyletic, co-circulating, and geographically associated [3]. NA EEEV, which has been associated with epizootics among horses and humans along the Atlantic and Gulf Coasts as well as in the Great Lakes Region, results in approximately 5–8 cases of neuroinvasive disease in humans yearly, while MADV has limited association with human disease, despite evidence of human exposure in endemic areas [4, 5]. NA EEEV is listed as a category B agent by the National Institute of Allergy and Infectious Diseases (NIAID) due to its virulence, its potential use as a biological weapon, and the lack of a licensed vaccine or effective antiviral treatment for human infections.

Though less well-studied than Venezuelan equine encephalitis virus, several small animal models have been used to study NA EEEV pathogenesis, including mice, hamsters, guinea pigs, and rats [6]. Mice are susceptible to NA EEEV induced mortality by a variety of routes of infection, including aerosol, intranasal, and subcutaneous in typically an age-dependent manner, with older mice becoming generally more resistant to infection [6]. However, systematic pathogenesis studies of EEEV infection in the mouse model are lacking.

Recently, NA EEEV strain FL93-939 was shown to be virulent in adult mice following peripheral infection [7]. Further, this strain was observed to decrease type I interferon induction in FL93-939-infected mice [7–9]. The purpose of this study was to characterize the pathogenesis of NA EEEV FL93-939 in mice after infection by one of three important routes: aerosol, intranasal, and subcutaneous, expecting that the pathogenesis would differ in a route-dependent manner. In an initial report, we described the clinical parameters of infection, including the effect of infection on body weight, temperature, and activity, and well as the virus distribution in tissues over time [10]. Here the histological and immunohistochemical features of infection by each of the three routes were examined. A complete understanding of the pathogenesis of NA EEEV and how it differs based on route of infection is an important step in the development of antiviral therapeutics.

## Results

Five mice from each time point were euthanized and perfused with 10 % NBF and routine tissues were collected for histologic and immunohistochemical analysis. There were many similarities in the pathogenesis of EEEV in the IN and AE studies and significant differences identified in the SC study, emphasizing the importance of evaluating various challenge routes when developing medical countermeasures.

### Intranasal challenge

**Fig. 1:**
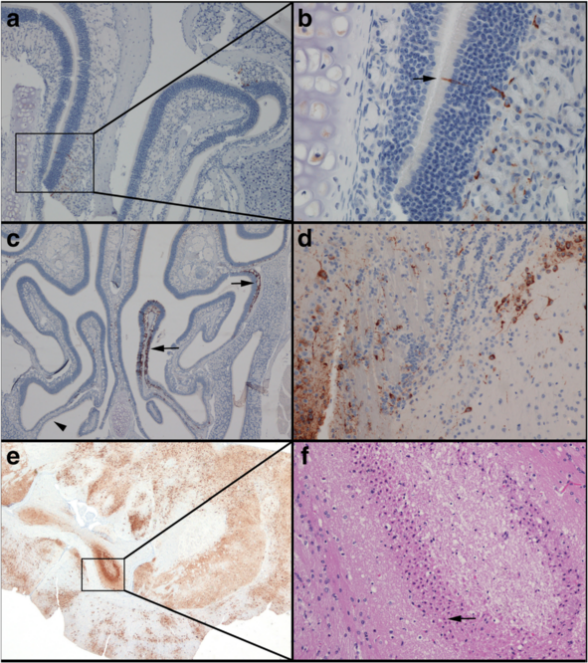
Immunohistochemical and histologic findings in mice after IN infection with EEEV strain FL93-939. Mice were infected with approximately 100LD_50_ and were euthanized at specified time points (n = 5). Viral antigen was first detected in the nasal cavity at1 dpi (box) (**a** magnification X100), specifically the olfactory epithelium and underlying lamina propria (arrow) (**b** magnification X400). A significant amount of viral antigen was present in the nasal cavity by 2 dpi, but was restricted to the olfactory epithelium (arrows); the respiratory epithelium was not involved (arrow head) (**c** magnification X40). Viral antigen was first detected in the olfactory bulb at 2 dpi (**d** magnification X400). Viral antigen was present throughout the brain by 5 dpi (**e** magnification X20) and there was significant neuronal cell death (hypereosinophilic, shrunken neurons with pyknosis or karyorrhexis, arrow) in the hippocampus (**f** magnification X200)

Key immunohistochemical findings and histologic lesions from the IN study are summarized in Tables 1 and 2, respectively. In the IN study, viral antigen was first detected in the nasal cavity (Fig. 1a), olfactory epithelium and lamina propria (Fig. 1b, arrow), and odontoblasts of a tooth as early as 1 dpi. However, no histologic lesions were noted at these sites at this time. By 2 dpi, viral antigen was observed more frequently in the nasal cavity (Fig. 1c), specifically in the olfactory epithelium and underlying lamina propria (Fig. 1c, arrows) and not within the respiratory epithelium (Fig. 1c, arrow head), as well as in the olfactory bulb (Fig. 1d), and in low numbers of neurons in the cerebrum, primarily in the piriform cortex. In some sites where antigen was present, single cell death was noted, characterized by pyknosis and eosinophilic cellular and karyorrhectic debris without inflammation, suggestive of apoptosis. Both the immunohistochemical and histologic findings at 3 dpi were similar to those seen at 2 dpi, with viral antigen present in the nasal cavity, olfactory bulb, and cerebrum, accompanied by single cell death in a small number of cells in the areas where antigen was present. At both 4 and 5 dpi; however, viral antigen was noted multifocally in the nasal cavity, and teeth, as well as diffusely throughout the olfactory bulb, frontal cortex, midbrain (Fig. 1e), cerebellum, brain stem, and spinal cord. Additionally, there was vacuolation, both intracytoplasmic (neuronal degeneration) and within the neuropil (spongiosis), within the olfactory bulb and spinal cord of some animals. Varying amounts of single cell death were noted in all sites where viral antigen was present, and a significant number of neurons in the hippocampus were apoptotic or absent (Fig. 1f , arrow). There was also mild leptomeningitis with occasional perivascular infiltrates (minimal encephalitis) in a few mice. At various early time points, viral antigen was present in the alveolar septa of the lung and sinus mononuclear cells of the mandibular lymph node, one of the draining lymph nodes of the nasal cavity. At later time points, viral antigen was also noted in ganglion cells of the retina (eye), osteoblasts and fibroblasts lining the trabecular bone surrounding the nasal cavity, pituitary gland, renal pelvic tubules (kidney), and myometrium (uterus) (Data not shown).

**Table 1:** Significant immunohistochemical findings observed in mice after IN challenge with EEEV

Tissue		6 hpi					12 hpi					1 dpi					2 dpi					3 dpi					4 dpi					5 dpi				
		6	7	8	9	10	6	7	8	9	10	6	7	8	9	10	6	7	8	9	10	6	7	8	9	10	6	7	8	9	10	6	7	8	9	10
Head	Nasal cavity	-	-	-	-	-	-	-	-	-	-	++	-	-	-	-	++	++	++	-	-	++	-	-	++	-	++	++	++	++	-	++	++	-	++	++
	Teeth	-	-	-	-	-	-	-	-	-	-	++	-	-	-	-	++	-	++	-	-	-	-	-	++	-	++	++	++	-	-	++	++	-	++	++
	Bone	-	-	-	-	-	-	-	-	-	-	-	-	-	-	-	-	++	-	-	-	++	-	-	++	-	-	-	-	-	-	-	-	-	-	-
	Eyes	-	-	-	-	-	-	-	-	-	-	-	-	-	-	-	-	-	-	-	-	-	-	-	-	-	-	++	-	-	-	++	++	-	-	-
Brain	Olfactory bulb	-	-	-	-	-	-	-	-	-	-	-	-	-	-	-	++	-	++	-	-	++	-	-	++	-	++	++	++	++	-	++	++	-	-	++
	Frontal cortex	-	-	-	-	-	-	-	-	-	-	-	-	-	-	-	++	-	-	-	-	++	-	-	++	-	++	++	++	++	-	++	++	-	-	++
	Midbrain	-	-	-	-	-	-	-	-	-	-	-	-	-	-	-	++	-	-	-	-	++	-	-	++	-	++	++	++	++	-	++	++	-	-	++
	Cerebellum	-	-	-	-	-	-	-	-	-	-	-	-	-	-	-	-	-	-	-	-	-	-	-	-	-	++	++	-	++	-	++	++	-	-	++
	Brain stem	-	-	-	-	-	-	-	-	-	-	-	-	-	-	-	-	-	-	-	-	-	-	-	-	-	++	++	-	++	-	++	++	-	-	++
	Pituitary gland	-	-	-	-	-	tnp	-	-	tnp	tnp	-	-	-	-	-	-	tnp	tnp	tnp	tnp	tnp	tnp	tnp	tnp	-	-	-	tnp	++	tnp	tnp	tnp	-	tnp	tnp
Spinal cord		-	-	-	-	-	-	-	-	-	-	-	-	-	-	-	-	-	-	-	-	++	-	-	-	-	++	++	-	++	-	++	++	-	-	++
Salivary gland	Mandibular	-	-	-	-	-	-	-	-	-	-	-	-	-	-	-	-	-	-	-	-	++	-	-	-	-	-	-	-	-	-	-	-	-	-	-
Haired skin		-	-	-	-	-	-	-	-	-	-	-	-	-	-	-	-	-	-	-	-	-	-	-	-	-	-	-	-	-	-	-	-	-	-	-
Lung		-	-	-	-	-	-	-	-	-	-	-	-	-	-	-	-	++	-	-	-	++	++	-	-	-	-	++	-	-	-	-	-	-	-	++
Heart		-	-	-	-	-	-	-	-	-	-	-	-	-	-	-	-	-	-	-	-	-	-	-	-	-	-	-	-	-	-	-	-	-	-	-
Spleen		-	-	-	-	-	-	-	-	-	-	-	-	-	-	-	-	-	-	-	-	-	-	-	-	-	-	-	-	-	-	-	-	-	-	-
Liver		-	-	-	-	-	-	-	-	-	-	-	-	-	-	-	-	-	-	-	-	-	-	-	-	-	-	-	-	-	-	-	-	-	-	-
Lymph node	Mandibular	-	-	-	-	-	-	-	-	-	-	-	-	-	-	-	-	++	-	-	++	-	-	-	-	-	-	-	++	++	-	++	-	-	-	-
	Tracheobronchial	-	-	-	-	-	-	-	-	-	-	-	-	-	-	-	-	-	-	-	-	-	-	-	-	tnp	tnp	-	-	-	-	-	-	-	-	-
	Axillary, left	-	tnp	-	-	-	-	-	-	-	-	-	-	-	-	-	-	-	-	tnp	-	-	-	-	-	tnp	-	-	-	-	-	tnp	tnp	tnp	-	-
	Axillary, right	-	-	-	-	-	tnp	-	-	-	-	-	-	-	-	-	-	tnp	-	tnp	-	-	tnp	tnp	tnp	-	-	-	-	-	tnp	tnp	tnp	tnp	-	tnp
	Mesenteric	-	-	-	-	-	-	-	-	-	-	-	-	-	-	-	-	-	-	-	-	-	-	-	-	-	-	-	-	-	-	-	-	-	-	-
	Inguinal, left	-	-	-	-	-	-	-	-	-	-	-	-	-	-	-	-	-	-	-	-	-	-	tnp	tnp	-	tnp	-	tnp	-	-	tnp	tnp	-	-	-
	Inguinal, right	-	tnp	tnp	tnp	tnp	tnp	-	tnp	tnp	tnp	-	-	-	-	-	-	-	-	-	-	-	-	-	tnp	-	-	-	-	-	tnp	-	-	tnp	-	tnp
	Popliteal, left	-	-	-	-	-	-	-	-	-	-	-	-	-	-	-	-	tnp	-	-	tnp	-	tnp	-	tnp	-	-	-	-	tnp	tnp	-	-	-	-	-
	Popliteal, right	-	-	-	-	tnp	-	-	-	tnp	-	-	-	-	-	-	-	-	-	-	-	-	-	tnp	-	-	-	-	-	-	tnp	tnp	tnp	tnp	-	-
Thymus		-	-	-	-	-	-	-	-	-	-	-	-	-	-	-	-	-	-	-	-	-	-	-	-	-	-	-	-	-	-	-	-	-	-	-
Thyroid gland		-	-	-	-	-	-	-	-	-	-	-	-	-	-	-	-	-	-	-	-	-	-	-	-	-	-	-	-	-	-	-	-	-	tnp	-
Pancreas		-	-	-	-	-	-	-	-	-	-	-	-	-	-	-	-	-	-	-	-	-	-	-	-	-	-	-	-	-	-	-	-	-	-	-
GI tract		-	-	-	-	-	-	-	-	-	-	-	-	-	-	-	-	-	-	-	-	-	-	-	-	-	-	-	-	-	-	-	-	-	-	-
Kidneys		-	-	-	-	-	-	-	-	-	-	-	-	-	-	-	-	-	-	-	-	-	-	-	-	-	-	-	-	-	-	++	-	-	-	-
Urinary bladder		-	-	tnp	tnp	-	-	-	-	-	-	-	-	-	-	-	-	-	-	-	-	-	-	-	-	-	-	-	-	-	-	-	-	-	-	-
Adrenal glands		-	-	-	-	-	-	-	-	-	-	-	-	-	-	-	-	-	-	-	-	-	-	-	-	-	-	-	-	-	-	-	-	-	-	-
Uterus	Myometrium	-	-	-	-	-	-	-	-	-	-	-	-	-	-	-	-	-	-	-	-	++	-	-	-	-	-	-	-	-	-	-	-	-	-	-
Ovaries		-	-	-	-	-	-	-	-	-	-	-	-	-	-	-	-	-	-	-	-	-	-	-	-	-	-	-	-	-	-	-	-	-	-	-
Rear leg, left		-	-	-	-	-	-	-	-	-	-	-	-	-	-	-	-	-	-	-	-	-	-	-	-	-	-	-	-	-	-	-	-	-	-	-
Rear foot, left		-	-	-	-	-	-	-	-	-	-	-	-	-	-	-	-	-	-	-	-	-	-	-	-	-	-	-	-	-	-	-	-	-	-	-
Rear leg, right		-	-	-	-	-	-	-	-	-	-	-	-	-	-	-	-	-	-	-	-	-	-	-	-	-	-	-	-	-	-	-	-	-	-	-
Rear foot, right		-	-	-	-	-	-	-	-	-	-	-	-	-	-	-	-	-	-	-	-	-	-	-	-	-	-	-	-	-	-	-	-	-	-	-

Symbols (++, ±, −) indicate that viral antigen was present and easily recognized (++); variably present throughout the tissue (±); or not detected histologically (−). Tnp = tissue not present on slide

**Table 2:** Significant histologic lesions noted in mice after IN challenge with EEEV

Tissue		6 hpi					12 hpi					1 dpi					2 dpi					3 dpi					4 dpi					5 dpi				
		6	7	8	9	10	6	7	8	9	10	6	7	8	9	10	6	7	8	9	10	6	7	8	9	10	6	7	8	9	10	6	7	8	9	10
Nasal cavity	Inflammation	-	-	-	-	-	-	-	-	-	-	-	-	-	-	-	-	-	-	-	-	-	-	-	-	-	-	-	-	-	-	-	-	-	-	-
	Single cell death	-	-	-	-	-	-	-	-	-	-	-	-	-	-	-	++	++	++	-	-	++	-	-	++	-	++	++	++	-	-	++	++	-	++	++
Tooth	Inflammation	-	-	-	-	-	-	-	-	-	-	-	-	-	-	-	-	-	-	-	-	-	-	-	-	-	-	-	-	-	-	-	-	-	-	-
	Single cell death	-	-	-	-	-	-	-	-	-	-	-	-	-	-	-	-	-	-	-	-	-	-	-	±	-	++	-	±	-	-	-	±	-	±	±
Bone	Single cell death	-	-	-	-	-	-	-	-	-	-	-	-	-	-	-	-	-	-	-	-	±	-	-	±	-	-	-	-	-	-	-	-	-	-	-
Eyes	Ganglion cell loss	-	-	-	-	-	-	-	-	-	-	-	-	-	-	-	-	-	-	-	-	-	-	-	-	-	-	-	-	-	-	±	-	-	-	-
Olfactory bulb	Vacuolation	-	-	-	-	-	-	-	-	-	-	-	-	-	±	-	±	-	-	-	-	-	-	-	-	-	-	-	-	++	-	++	++	-	-	++
	Single cell death	-	-	-	-	-	-	-	-	-	-	-	-	-	-	-	-	-	-	-	-	±	-	-	±	-	++	±	±	++	-	++	++	-	-	++
Cerebrum	Inflammation	-	-	-	-	-	-	-	-	-	-	-	-	-	-	-	-	-	-	-	-	-	-	-	-	-	-	-	-	2mf	-	-	2mf	-	-	2mf
	Single cell death	-	-	-	-	-	-	-	-	-	-	-	-	-	-	-	-	-	-	-	-	±	-	-	±	-	++	±	±	++	-	++	++	-	-	++
Cerebellum	Inflammation	-	-	-	-	-	-	-	-	-	-	-	-	-	-	-	-	-	-	-	-	-	-	-	-	-	-	-	-	-	-	-	-	-	-	-
	Single cell death	-	-	-	-	-	-	-	-	-	-	-	-	-	-	-	-	-	-	-	-	-	-	-	-	-	++	±	-	±	-	++	++	-	-	++
Brain stem	Inflammation	-	-	-	-	-	-	-	-	-	-	-	-	-	-	-	-	-	-	-	-	-	-	-	-	-	-	-	-	-	-	-	-	-	-	-
	Single cell death	-	-	-	-	-	-	-	-	-	-	-	-	-	-	-	-	-	-	-	-	-	-	-	-	-	-	-	-	-	-	-	-	-	-	++
Spinal Cord	Vacuolation	-	-	-	-	-	-	-	-	-	-	-	-	-	-	-	-	-	-	-	-	-	-	-	-	-	-	-	-	-	-	-	-	-	-	++
	Single cell death	-	-	-	-	-	-	-	-	-	-	-	-	-	-	-	-	-	-	-	-	-	-	-	-	-	-	±	-	-	-	++	++	-	-	±
Lung	Hemorrhage	-	-	-	-	-	±	-	±	±	-	-	±	±	±	-	±	±	-	-	-	±	-	-	-	±	±	±	-	-	±	±	±	±	±	±
	Inflammation	-	-	-	-	-	-	-	-	-	-	-	-	-	-	-	-	-	-	-	2mf	-	-	2mf	2mf	-	-	-	2mf	-	-	-	-	-	-	-
	Single cell death	-	-	-	-	-	-	-	-	-	-	-	-	-	-	-	-	±	-	-	-	±	±	-	-	-	-	±	-	-	-	-	-	-	-	-
Repro tract	Single cell death	-	-	-	-	-	-	-	-	-	-	-	-	-	-	-	-	-	-	-	-	±	-	-	-	-	-	-	-	-	-	-	-	-	-	-

Symbols (++, ±, −) indicate whether the lesion was present and easily recognized (++); variably present throughout the tissue (±); or not detected histologically (−). A score of 1–5 indicates the severity of the inflammation: 1 (minimal); 2 (mild); 3 (moderate); 4 (marked); 5 (severe). Distribution of the lesion: f (focal); fe (focally extensive); mf (multifocal); d (diffuse)

### Aerosol challenge

**Fig. 2:**
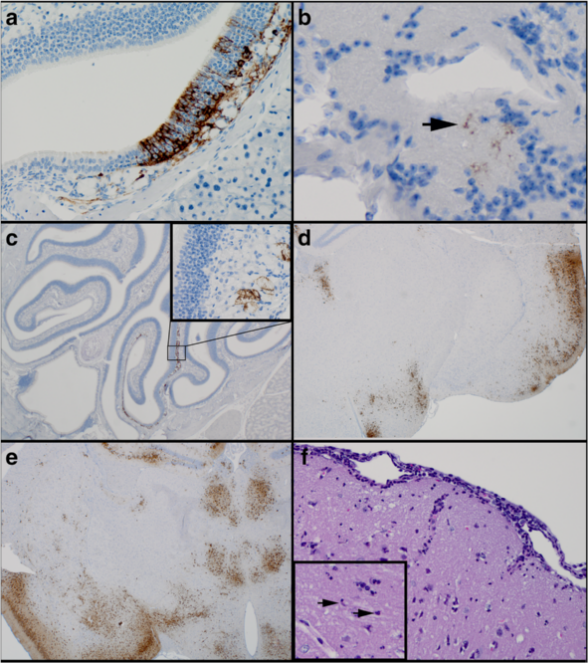
Immunohistochemical and histologic findings in mice after AE infection with EEEV strain FL93-939. Mice were infected with approximately 100LD_50_ and were euthanized at specified time points (n = 5, except 4dpi n = 10). Viral antigen was first detected in the olfactory nasal epithelium, lamina propria, and olfactory nerve (**a** magnification X200), as well as the olfactory bulb at 1 dpi (**b** magnification X200). Multifocally, viral antigen was present in the nasal cavity (**c** magnification X20), the olfactory nerve (**c**; inset, magnification X400), and cerebrum, especially the piriform cortex, by 2 dpi (**d** magnification X20). Viral antigen was present throughout the brain by 3 dpi (**e** magnification X20) and there was meningoencephalitis in the cerebrum (**f** magnification X200) and multifocal neuronal cell death (hypereosinophilic, shrunken neurons with pyknosis or karyorrhexis; inset, arrow)

Significant immunohistochemical findings and histologic lesions from the AE study are summarized in Tables 3 and 4, respectively. The findings in the AE study were similar to IN; however, there were some important differences. Analogous to the IN study, viral antigen was first detected in the olfactory epithelium and lamina propria of the nasal cavity at 1 dpi (Fig. 2a). However, unlike the IN study, it was also present in the olfactory nerve and the olfactory bulb (Fig. 2b) at 1 dpi; the first indication of neural invasion. By 2 dpi, unlike the IN study, in the AE study, viral antigen was present in the majority of mice within the nasal cavity (Fig. 2c), the olfactory nerve (Fig. 2c, inset), teeth (odontoblasts) and prominently within the olfactory bulb and/or brain, primarily within the piriform cortex (Fig. 2d) and sporadically within the thalamus, with. At 3 dpi, viral antigen was present in the nasal cavity, olfactory bulb, frontal cortex, and midbrain of all mice, and within the brain stem of 2 animals. Single cell death was easily recognized within the nasal cavity, olfactory bulb, and cerebrum and was variably present in other areas where viral antigen was located. Within the brain, there were variable amounts of neuronal vacuolation, spongiosis, and moderate meningoencephalitis in some mice. This arm of the study was terminated on 4 dpi because a majority of remaining mice had severe clinical disease. Therefore, the 4 dpi group consisted of 10 mice. In 9 of these mice, viral antigen was multifocally to diffusely present throughout nasal cavity, olfactory bulbs, frontal cortex, midbrain (Fig. 2e), cerebellum, and brain stem, as well as the spinal cord, lung and pituitary gland. Again, single cell death without inflammation (apoptosis) was easily recognized in all tissues, including the cerebrum (Fig. 2f, arrow), in which viral antigen was present. Similar to the lesions noted at 3 dpi, there was variable neuronal vacuolation, spongiosis, and moderate meningoencephalitis (Fig. 2f) in the brain of some mice. *In situ* hybridization results correlated with the distribution of viral antigen (data not shown). Similar to the findings in the IN study, viral antigen was variably present after 1 dpi in the lung (alveolar septa, adjacent to terminal bronchioles), eye (ganglion cells of the retina), reproductive tract (myometrium and/or ovary), and renal pelvic epithelium (Data not shown).

**Table 3:** Significant immunohistochemical findings observed in mice after AE challenge with EEEV

Tissue		6 hpi					12 hpi					1 dpi					2 dpi					3 dpi					4 dpi					5 dpi				
		6	7	8	9	10	6	7	8	9	10	6	7	8	9	10	6	7	8	9	10	6	7	8	9	10	6	7	8	9	10	6	7	8	9	10
Head	Nasal cavity	-	-	-	-	-	-	-	-	-	-	-	++	++	-	++	++	++	-	++	++	++	++	++	++	++	++	++	++	++	-	++	++	++	++	++
	Teeth	-	-	-	-	-	-	-	-	-	-	-	-	-	-	-	++	++	-	-	++	++	++	-	-	++	++	++	++	-	-	++	++	++	++	++
	Bone	-	-	-	-	-	-	-	-	-	-	-	-	-	-	-	-	-	-	-	-	-	-	-	-	-	-	-	-	-	-	-	-	-	-	-
	Eyes	-	-	-	-	-	-	-	-	-	-	-	-	-	-	-	-	-	-	-	-	-	-	-	-	-	++	++	++	-	-	-	-	-	++	++
Brain	Olfactory bulb	-	-	-	-	-	-	-	-	-	-	-	-	-	-	++	++	++	-	++	-	++	++	++	++	++	++	++	++	++	-	++	-	++	++	++
	Frontal cortex	-	-	-	-	-	-	-	-	-	-	-	-	-	-	-	++	++	-	++	-	++	++	++	++	++	++	++	++	++	-	++	++	++	++	++
	Midbrain	-	-	-	-	-	-	-	-	-	-	-	-	-	-	-	++	-	-	-	-	++	++	++	++	++	++	++	++	++	-	++	++	++	++	++
	Cerebellum	-	-	-	-	-	-	-	-	-	-	-	-	-	-	-	-	-	-	-	-	-	-	-	-	-	++	++	++	-	-	++	++	++	++	++
	Brain stem	-	-	-	-	-	-	-	-	-	-	-	-	-	-	-	-	-	-	-	-	++	-	++	-	tnp	++	++	++	++	-	++	++	++	++	++
	Pituitary gland	-	tnp	-	-	tnp	-	tnp	-	-	tnp	-	-	-	-	-	++	-	-	-	-	-	-	-	-	++	++	++	++	-	-	-	tnp	tnp	++	++
Spinal cord		-	-	-	-	-	-	-	-	-	-	-	-	-	-	-	-	-	-	-	-	-	-	-	-	++	++	++	++	-	-	++	++	++	++	++
Salivary gland	Mandibular	-	-	-	-	-	-	-	-	-	-	-	-	-	-	-	-	-	-	-	-	-	-	-	-	-	-	-	-	-	-	-	-	-	-	-
Haired skin		-	-	-	-	-	-	-	-	-	-	-	-	-	-	-	-	-	-	-	-	-	-	-	-	-	-	-	-	-	-	-	-	-	-	-
Lung		-	-	-	-	-	-	-	-	-	-	-	-	-	-	-	++	++	-	-	-	++	++	-	++	++	++	++	++	++	++	++	++	++	++	++
Heart		-	-	-	-	-	-	-	-	-	-	-	-	-	-	-	-	-	-	-	-	-	-	-	-	-	-	-	-	-	-	-	++	-	-	-
Spleen		-	-	-	-	-	-	-	-	-	-	-	-	-	-	-	-	-	-	-	-	-	-	-	-	-	-	-	-	-	-	-	-	-	-	-
Liver		-	-	-	-	-	-	-	-	-	-	-	-	-	-	-	-	-	-	-	-	-	-	-	-	-	-	-	-	-	-	-	-	-	-	-
Lymph node	Mandibular	-	-	-	-	-	-	-	-	-	-	-	-	-	-	-	tnp	-	-	-	-	-	-	-	-	-	-	-	-	-	-	-	-	-	-	-
	Tracheobronchial	-	-	-	-	-	-	-	-	-	-	-	-	-	-	-	-	-	-	-	-	-	-	-	-	-	-	-	-	-	-	-	-	-	-	-
	Axillary, left	-	-	tnp	-	tnp	-	-	-	-	tnp	-	-	tnp	-	-	-	-	-	-	-	-	-	-	-	-	-	-	-	-	-	-	-	-	-	-
	Axillary, right	-	-	-	-	-	-	-	-	-	-	-	-	-	-	-	tnp	-	-	-	-	-	-	-	-	-	-	-	-	-	tnp	-	-	-	-	-
	Mesenteric	-	-	-	-	-	-	-	-	-	-	-	-	-	-	-	-	-	-	-	-	-	-	-	-	-	-	-	-	-	-	-	-	-	-	-
	Inguinal, left	-	-	-	-	-	-	-	-	-	-	-	-	tnp	-	-	-	-	tnp	tnp	tnp	-	-	-	-	-	-	tnp	-	tnp	tnp	-	-	-	-	-
	Inguinal, right	-	tnp	tnp	-	-	-	-	-	-	-	-	-	-	-	-	-	-	-	tnp	-	-	-	-	-	-	tnp	tnp	tnp	-	tnp	-	-	-	-	-
	Popliteal, left	-	-	-	tnp	-	tnp	tnp	-	tnp	-	tnp	-	tnp	-	-	-	tnp	tnp	-	tnp	-	tnp	tnp	-	-	-	-	-	-	-	-	-	-	tnp	tnp
	Popliteal, right	tnp	-	tnp	-	-	tnp	tnp	-	tnp	tnp	tnp	tnp	-	-	-	-	-	-	-	tnp	tnp	-	tnp	-	tnp	tnp	-	-	tnp	-	-	-	-	-	-
Thymus		-	-	-	-	-	-	-	-	-	-	-	-	-	-	-	-	-	-	-	-	-	-	-	-	-	-	-	-	-	-	-	-	-	-	-
Thyroid gland		tnp	-	-	-	-	-	tnp	-	tnp	tnp	tnp	-	-	-	-	-	-	tnp	tnp	-	tnp	tnp	-	tnp	tnp	-	tnp	tnp	tnp	tnp	-	tnp	tnp	-	-
Pancreas		-	-	-	-	-	-	-	-	-	-	-	-	-	-	-	-	-	-	-	-	tnp	-	-	-	-	-	-	-	-	-	-	-	-	-	-
GI tract		-	-	-	-	-	-	-	-	-	-	-	-	-	-	-	-	-	-	-	-	-	-	-	-	-	-	-	-	-	-	-	-	-	-	-
Kidneys		-	-	-	-	-	-	-	-	-	-	-	-	-	-	-	-	-	-	-	-	-	-	-	-	-	-	-	-	-	-	-	-	-	-	++
Urinary bladder		-	-	-	-	-	-	-	-	-	-	-	-	-	-	-	-	-	-	-	-	-	-	-	-	-	-	-	-	-	-	-	-	-	-	-
Adrenal glands		-	-	-	-	-	-	-	-	-	-	-	-	-	-	-	-	-	-	-	-	-	-	-	-	-	-	-	-	-	-	-	-	-	-	-
Uterus	Myometrium	-	-	-	-	-	-	-	-	-	-	-	-	-	-	-	++	-	-	-	++	-	-	-	-	++	-	-	++	++	++	-	++	-	-	-
Ovaries		-	-	-	-	-	-	-	-	-	-	-	-	-	-	-	++	++	-	-	++	-	-	++	-	-	-	-	-	-	-	-	++	-	-	-
Rear leg, left		-	-	-	-	-	-	-	-	-	-	-	-	-	-	-	-	-	-	-	-	-	-	-	-	-	-	-	-	-	-	-	-	-	-	-
Rear foot, left		-	-	-	-	-	-	-	-	-	-	-	-	-	-	-	-	-	-	-	-	-	-	-	-	-	-	-	-	-	-	-	-	-	-	-
Rear leg, right		-	-	-	-	-	-	-	-	-	-	-	-	-	-	-	-	-	-	-	-	-	-	-	-	-	-	-	-	-	-	-	-	-	-	-
Rear foot, right		-	-	-	-	-	-	-	-	-	-	-	-	-	-	-	-	-	-	-	-	-	-	-	-	-	-	-	-	-	-	-	-	-	-	-

Symbols (++, ±, −) indicate that viral antigen was present and easily recognized (++); variably present throughout the tissue (±); or not detected histologically (−). Tnp = tissue not present on slide

**Table 4:** Significant histologic lesions noted in mice after AE challenge with EEEV

Tissue		6 hpi					12 hpi					1 dpi					2 dpi					3 dpi					4 dpi					5 dpi				
		6	7	8	9	10	6	7	8	9	10	6	7	8	9	10	6	7	8	9	10	6	7	8	9	10	6	7	8	9	10	6	7	8	9	10
Nasal cavity	Inflammation	-	-	-	-	-	-	-	-	-	-	-	-	-	-	-	-	-	-	3mf	-	3fe	-	-	3fe	-	-	-	-	-	-	3mf	-	-	3fe	3mf
	Single cell death	-	-	-	-	-	-	-	-	-	-	-	++	±	-	±	++	++	-	++	++	++	++	++	++	++	++	++	++	++	-	++	++	++	++	++
Tooth	Inflammation	-	-	-	-	-	-	-	-	-	-	-	-	-	-	-	-	-	-	-	-	-	-	-	-	-	-	-	-	-	-	-	-	-	-	-
	Single cell death	-	-	-	-	-	-	-	-	-	-	-	-	-	-	-	-	-	-	-	±	±	±	-	±	±	±	±	±	-	-	±	±	±	-	±
Bone	Single cell death	-	-	-	-	-	-	-	-	-	-	-	-	-	-	-	-	-	-	-	-	-	-	-	-	-	-	-	-	-	-	-	-	-	-	-
Eyes	Ganglion cell loss	-	-	-	-	-	-	-	-	-	-	-	-	-	-	-	-	-	-	-	-	-	-	-	-	-	-	-	-	-	-	-	-	-	-	-
Olfactory bulb	Vacuolation	-	-	-	-	-	-	-	-	-	-	-	-	-	-	-	-	±	-	-	-	-	±	±	-	±	±	-	±	-	-	±	-	±	±	±
	Single cell death	-	-	-	-	-	-	-	-	-	-	-	-	-	-	-	±	±	-	-	-	++	++	++	++	++	++	++	++	++	-	++	-	++	++	++
Cerebrum	Inflammation	-	-	-	-	-	-	-	-	-	-	-	-	-	-	-	-	-	-	-	-	-	-	-	-	++	±	±	±	-	-	++	-	++	±	±
	Single cell death	-	-	-	-	-	-	-	-	-	-	-	-	-	-	-	-	-	-	-	-	-	-	++	-	-	-	-	-	-	-	-	-	-	-	-
Cerebellum	Inflammation	-	-	-	-	-	-	-	-	-	-	-	-	-	-	-	-	-	-	-	-	3d	-	2mf	-	4mf	-	-	3d	3d	-	3d	-	3d	-	3mf
	Single cell death	-	-	-	-	-	-	-	-	-	-	-	-	-	-	-	±	-	-	-	-	++	±	++	±	++	++	++	++	++	-	++	-	++	++	++
Brain stem	Inflammation	-	-	-	-	-	-	-	-	-	-	-	-	-	-	-	-	-	-	-	-	-	-	-	-	-	-	-	-	-	-	-	-	-	-	-
	Single cell death	-	-	-	-	-	-	-	-	-	-	-	-	-	-	-	-	-	-	-	-	±	-	±	-	-	±	±	±	±	-	±	±	±	±	±
Spinal Cord	Vacuolation	-	-	-	-	-	-	-	-	-	-	-	-	-	-	-	-	-	-	-	-	-	-	-	-	-	-	-	-	-	-	-	-	-	3mf	-
	Single cell death	-	-	-	-	-	-	-	-	-	-	-	-	-	-	-	-	-	-	-	-	±	-	±	-	-	-	-	-	-	-	±	±	±	±	±
Lung	Hemorrhage	-	-	-	-	-	-	-	-	-	-	-	-	-	-	-	-	-	-	-	-	-	-	-	-	-	±	-	-	-	-	-	-	-	-	-
	Inflammation	-	-	-	-	-	-	-	-	-	-	-	-	-	-	-	-	-	-	-	-	-	-	-	-	-	±	±	±	-	-	±	-	±	±	±
	Single cell death	-	-	-	-	-	-	-	-	-	-	±	-	±	-	-	-	-	-	-	-	-	-	-	-	-	-	±	±	-	-	-	-	±	-	-
Repro tract	Single cell death	-	-	-	-	-	-	-	-	-	-	-	-	-	-	-	2fe	-	-	-	-	2fe	-	-	2mf	-	-	-	-	-	-	-	-	-	-	-

Symbols (++, ±, −) indicate whether the lesion was present and easily recognized (++); variably present throughout the tissue (±); or not detected histologically (−). A score of 1–5 indicates the severity of the inflammation: 1 (minimal); 2 (mild); 3 (moderate); 4 (marked); 5 (severe). Distribution of the lesion: f (focal); fe (focally extensive); mf (multifocal); d (diffuse)

### Subcutaneous challenge

**Fig. 3:**
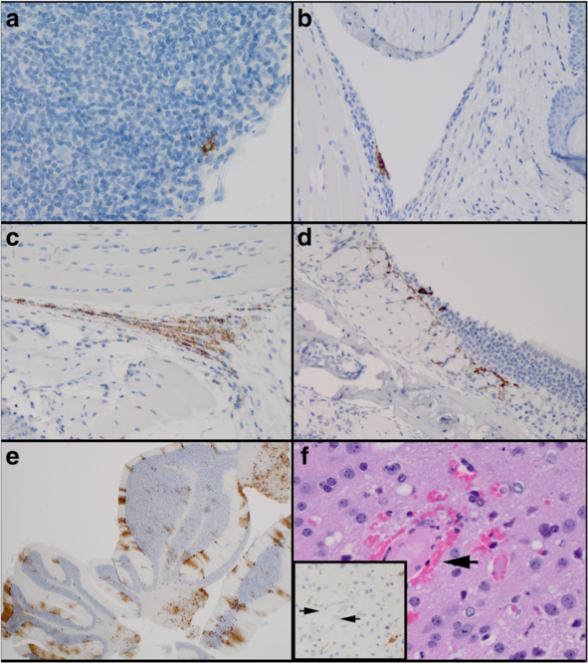
Immunohistochemical and histologic findings in mice after SC (left footpad) infection with EEEV strain FL93-939. Mice were infected with approximately 30LD_50_ and were euthanized at specified time points (n = 5). Viral antigen was first detected in the left popliteal (draining) lymph node at 6 hpi (**a** magnification X400). At the inoculation site, viral antigen was present in synovial cells at 12 hpi (**b** magnification X200). Viral antigen was found in numerous fibroblasts in the left foot at 2 dpi (**c** magnification X200). Viral antigen was first detected in the olfactory epithelium at 3 dpi (**d** magnification X200) and was present throughout the brain, including the cerebellum by 4 dpi (**e** magnification X20). Within the cerebrum there was meningoencephalitis with vasculitis, thrombosis, hemorrhage (arrows), and spongiosis by 6 dpi (**f** magnification X400); however, viral antigen was not present around thrombotic vessels (inset, between arrows)

Significant immunohistochemical findings and histologic lesions from the SC study are summarized in Tables 5 and 6, respectively. The results of the SC (left footpad) study were quite different than the IN and AE studies. As expected, viral antigen was present at the inoculation site (left footpad) from 6 hpi, and remained within the left foot to varying degrees throughout the study. Viral antigen was noted in the left popliteal lymph node, the draining lymph node of the foot and lower leg, from 6 hpi (Fig. 3a) through 2 dpi. Cells most commonly positive for viral antigen in the foot included synovial cells (Fig. 3b), connective tissue fibroblasts (Fig. 3c), and skeletal myocytes; however, antigen was also present in and around hair follicles, within the epidermis and mononuclear inflammatory cells near the inoculation site. No significant histologic lesions were noted in the left foot until 1 dpi when scattered single cell death was apparent. Single cell death (apoptosis), cellulitis, and myocyte degeneration, necrosis and regeneration were noted in most mice, to varying degrees, in the left foot from 2 dpi through the end of the study (Data not shown). Similar to the results of the IN study, in the lymph node, viral antigen was only found in histocytes and/or cells with morphologic features of dendritic cells within the subcapsular sinus, not within the follicles of the cortex. Unlike the IN and AE studies where viral antigen was first noted in the nasal cavity olfactory epithelium and lamina propria at 1 dpi, in the SC study this occurred 2 days later, at 3 dpi (Fig. 3d) and was detected in a low number of mice through 7 dpi. From 4 dpi through 8 dpi, viral antigen was detected in either the olfactory epithelium/lamina propria and/or the teeth in only 8 of 25 mice; viral antigen was also detected in the olfactory bulb or cerebrum in 7 of those 8 animals, 4 of which had viral antigen throughout the cerebrum, cerebellum (Fig. 3e), and brain stem. Significantly, these 4 mice also had moderate-marked neuronal apoptosis, spongiosis, meningoencephalitis, as well as vasculitis, thrombosis, and perivascular hemorrhage (Fig. 3f, arrows), which was not seen in either the IN or AE studies. Interestingly, viral antigen was not closely associated with the areas of vasculitis or thrombosis (Fig. 3f, inset, between arrows). In one mouse at 7 dpi, viral antigen was detected throughout the brain, but was not detected in either the nasal cavity or teeth. However, histologically there was a significant mucosal hyperplastic response along with a marked secondary bacterial rhinitis.

**Table 5:** Significant immunohistochemical findings observed in mice after SC challenge with EEEV

Tissue		6 hpi				12 hpi					1 dpi					2 dpi					3 dpi					4 dpi					5 dpi					6 dpi					7 dpi					8 dpi				
		6	7	8	9	6	7	8	9	10	6	7	8	9	10	6	7	8	9	10	6	7	8	9	10	6	7	8	9	10	6	7	8	9	10	6	7	8	9	10	6	7	8	9	10	6	7	8	9	10
Head	Nasal cavity	-	-	-	-	-	-	-	-	-	-	-	-	-	-	-	-	-	-	-	-	-	-	-	++	-	-	++	-	-	-	-	-	-	++	-	-	-	++	++	-	-	-	-	++	-	-	-	-	-
	Teeth	-	-	-	-	-	-	-	-	-	-	-	-	-	-	-	-	-	-	-	-	-	-	-	++	-	++	++	++	-	-	-	-	-	-	-	-	-	-	-	-	-	-	-	-	-	-	-	-	-
	Bone	-	-	-	-	-	-	-	-	-	-	-	-	-	-	-	-	-	-	-	-	-	-	-	-	-	-	-	-	-	-	-	-	-	-	-	-	-	-	-	-	-	-	-	-	-	-	-	-	-
	Eyes	-	-	-	-	-	-	-	-	-	-	-	-	-	-	-	-	-	-	-	-	-	-	-	-	-	-	-	-	-	-	-	-	-	-	-	-	-	++	-	++	-	-	-	-	-	-	-	-	-
Brain	Olfactory bulb	-	-	-	-	-	-	-	-	-	-	-	-	-	-	-	-	-	-	-	-	-	-	-	-	-	++	++	-	-	-	-	-	-	++	-	-	-	++	++	++	-	-	-	++	-	-	-	-	-
	Frontal cortex	-	-	-	-	-	-	-	-	-	-	-	-	-	-	-	-	-	-	-	-	-	-	-	-	-	-	++	++	-	-	-	-	-	-	-	-	-	++	++	++	-	-	-	++	-	-	-	-	-
	Midbrain	-	-	-	-	-	-	-	-	-	-	-	-	-	-	-	-	-	-	-	-	-	-	-	-	-	-	++	-	-	-	-	-	-	-	-	-	-	++	++	++	-	-	-	++	-	-	-	-	-
	Cerebellum	-	-	-	-	-	-	-	-	-	-	-	-	-	-	-	-	-	-	-	-	-	-	-	-	-	-	++	-	-	-	-	-	-	-	-	-	-	++	++	++	-	-	-	++	-	-	-	-	-
	Brain stem	-	-	-	-	-	-	-	-	-	-	-	-	-	-	-	-	-	-	-	-	-	-	-	-	-	-	++	-	-	-	-	-	-	-	-	-	-	++	++	++	-	-	-	++	-	-	-	-	-
	Pituitary gland	-	-	tnp	-	-	-	tnp	-	-	-	tnp	-	tnp	-	-	-	-	tnp	-	-	-	-	-	-	tnp	tnp	tnp	tnp	-	-	-	tnp	tnp	-	-	tnp	-	-	-	-	-	tnp	-	++	-	-	++	tnp	-
Spinal cord		-	-	-	-	-	-	-	-	-	-	-	-	-	-	-	-	-	-	-	-	-	-	-	-	-	-	-	-	-	-	-	-	-	-	-	-	-	++	++	++	-	-	-	++	-	-	-	-	-
Salivary gland	Mandibular	-	-	-	-	-	-	tnp	-	-	-	-	-	-	-	-	-	-	-	-	-	-	-	-	-	-	-	-	-	-	-	-	-	-	-	-	-	-	-	-	-	-	-	-	-	-	-	-	-	-
Haired skin		-	-	-	-	-	-	-	-	-	-	-	-	-	-	-	-	-	-	-	-	-	-	-	-	-	-	-	-	-	-	-	-	-	-	-	-	-	-	-	-	-	-	-	-	-	-	-	-	-
Lung		-	-	-	-	-	-	-	-	-	-	-	-	-	-	-	-	-	-	-	-	-	-	-	-	-	-	-	-	-	-	-	-	-	-	-	-	-	-	-	-	-	-	-	-	-	-	-	-	-
Heart		-	-	-	-	-	-	-	-	-	-	-	-	-	-	-	-	-	-	-	-	-	-	-	-	-	-	-	-	-	-	-	-	-	-	-	-	-	-	-	-	-	-	-	-	-	-	-	-	-
Spleen		-	-	-	-	-	-	-	-	-	-	-	-	-	-	-	-	-	-	-	-	-	-	-	-	-	-	-	-	-	-	-	-	-	-	-	-	-	-	-	-	-	-	-	-	-	-	-	-	-
Liver		-	-	-	-	-	-	-	-	-	-	-	-	-	-	-	-	-	-	-	-	-	-	-	-	-	-	-	-	-	-	-	-	-	-	-	-	-	-	-	-	-	-	-	-	-	-	-	-	-
Lymph node	Mandibular	-	-	-	-	-	-	-	-	-	-	-	-	-	-	-	-	-	-	-	-	-	++	-	-	-	-	-	-	-	-	-	-	-	++	-	-	-	-	-	-	-	-	-	-	-	-	-	-	tnp
	Tracheobronchial	-	-	-	-	-	-	-	-	-	-	-	-	-	-	-	-	-	-	-	-	tnp	-	tnp	-	-	-	-	-	-	-	-	-	-	-	-	-	-	-	-	-	-	-	-	-	-	-	tnp	-	-
	Axillary, left	-	-	tnp	-	-	-	-	tnp	-	-	-	tnp	-	-	-	-	-	-	-	tnp	-	tnp	-	-	-	-	-	-	-	-	-	-	-	-	-	tnp	-	-	-	-	-	-	-	tnp	-	-	-	-	-
	Axillary, right	-	-	-	-	-	-	-	-	tnp	-	-	-	tnp	-	tnp	-	-	-	-	-	-	-	tnp	-	-	-	-	-	-	-	-	-	tnp	-	-	-	-	-	-	-	-	-	tnp	-	-	-	-	-	-
	Mesenteric	-	-	-	-	-	-	-	-	-	-	-	-	-	-	-	-	-	-	-	-	-	-	-	-	-	-	-	-	-	-	-	-	-	-	-	-	-	-	-	-	-	-	-	-	-	-	-	-	-
	Inguinal, left	-	-	-	-	-	-	-	-	-	-	-	-	-	-	tnp	-	tnp	-	-	-	-	-	-	-	-	-	-	-	-	-	-	-	-	-	-	-	-	-	-	-	-	-	-	-	-	-	-	-	tnp
	Inguinal, right	tnp	-	tnp	tnp	tnp	tnp	-	tnp	-	-	-	tnp	tnp	tnp	tnp	-	-	-	-	-	-	-	-	tnp	-	-	-	-	-	-	-	-	-	-	-	-	-	-	-	-	-	-	-	-	-	-	-	-	tnp
	Popliteal, left	++	-	-	tnp	++	tnp	++	++	++	++	tnp	tnp	++	++	tnp	++	-	-	-	-	tnp	-	-	-	-	-	-	-	-	-	-	-	-	-	-	-	-	-	-	-	-	-	-	-	-	-	-	-	-
	Popliteal, right	-	-	-	tnp	tnp	-	-	tnp	-	-	+++	tnp	tnp	tnp	tnp	-	-	-	-	-	-	-	-	-	-	-	-	-	tnp	tnp	-	-	-	-	-	-	-	-	-	-	-	tnp	-	tnp	tnp	-	-	-	-
Thymus		-	-	-	-	-	-	-	-	-	-	-	-	-	tnp	-	-	-	-	-	-	-	-	-	-	-	tnp	-	-	-	-	-	-	-	-	-	-	-	-	-	-	-	-	-	-	-	-	-	-	-
Thyroid gland		tnp	tnp	-	-	-	-	-	-	tnp	-	tnp	-	-	tnp	tnp	tnp	-	tnp	-	-	-	-	-	-	tnp	tnp	-	-	tnp	-	-	-	-	-	tnp	-	-	-	tnp	-	tnp	tnp	-	tnp	-	tnp	tnp	-	-
Pancreas		-	-	-	-	-	-	-	-	-	-	-	-	-	-	-	-	-	-	-	-	-	-	-	-	-	-	-	-	-	-	-	-	-	-	-	-	-	-	-	-	-	-	-	-	-	-	-	-	-
GI tract		-	-	-	-	-	-	-	-	-	-	-	-	-	-	-	-	-	-	-	-	-	-	-	-	-	-	-	-	-	-	-	-	-	-	-	-	-	-	-	-	-	-	-	-	-	-	-	-	-
Kidneys		-	-	-	-	-	-	-	-	-	-	-	-	-	-	-	-	-	-	-	-	-	-	-	-	-	-	-	-	-	-	-	-	-	-	-	-	-	-	-	-	-	-	-	-	-	-	-	-	-
Urinary bladder		-	-	-	-	-	-	-	-	-	-	-	-	-	-	-	-	-	-	-	-	-	-	-	-	-	-	-	-	-	-	-	-	-	-	-	-	-	-	-	-	-	-	-	-	-	-	tnp	-	-
Adrenal glands		-	-	-	-	-	-	-	-	-	-	-	-	-	-	-	-	-	-	-	-	-	-	-	-	-	-	-	-	-	-	-	-	-	-	-	-	-	-	-	-	-	-	-	-	-	-	-	-	tnp
Uterus	Myometrium	-	-	-	-	-	-	-	-	-	-	-	-	-	-	-	-	-	-	-	-	-	-	-	-	-	-	-	-	-	-	-	-	-	-	-	-	-	-	-	-	-	-	-	-	-	-	-	-	-
Ovaries		-	-	-	-	-	-	-	-	-	-	-	-	-	-	-	-	-	-	-	-	-	-	-	tnp	-	-	-	-	-	-	-	tnp	-	-	-	-	-	-	-	-	-	-	-	-	-	-	-	-	-
Rear leg, left		-	-	-	-	-	-	-	-	-	-	-	-	-	-	-	-	-	-	-	-	-	-	-	-	-	-	-	-	-	-	-	-	-	-	-	-	-	-	-	-	-	-	-	-	-	-	-	-	-
Rear foot, left		++	-	-	-	++	++	++	++	++	++	++	++	++	++	++	++	++	++	++	++	++	++	++	++	++	++	++	++	++	++	++	++	++	++	++	++	++	++	++	++	++	++	++	++	++	-	-	++	-
Rear leg, right		-	-	-	-	-	-	-	-	-	-	-	-	-	-	-	-	-	-	-	-	-	-	-	-	-	-	-	-	-	-	-	-	-	-	-	-	-	-	-	-	-	-	-	-	-	-	-	-	-
Rear foot, right		-	-	-	-	-	-	-	-	-	-	-	-	-	-	-	-	-	-	-	-	-	-	-	-	-	-	-	-	-	-	-	-	-	-	-	-	-	-	-	-	-	-	-	-	-	-	-	-	-

Symbols (++, ±, −) indicate that viral antigen was present and easily recognized (++); variably present throughout the tissue (±); or not detected histologically (−). Tnp = tissue not present on slide

**Table 6:** Significant histologic lesions noted in mice after SC challenge with EEEV

Tissue		6 hpi				12 hpi					1 dpi					2 dpi					3 dpi					4 dpi					5 dpi					6 dpi					7 dpi					8 dpi				
		6	7	8	9	6	7	8	9	10	6	7	8	9	10	6	7	8	9	10	6	7	8	9	10	6	7	8	9	10	6	7	8	9	10	6	7	8	9	10	6	7	8	9	10	6	7	8	9	10
Nasal cavity	Inflammation	-	-	-	-	-	-	-	-	-	-	-	-	-	-	-	-	-	-	-	-	-	-	-	-	-	-	-	-	-	-	-	-	-	-	-	-	-	5mf	5mf	5mf	-	-	-	3mf	-	-	-	-	-
	Single cell death	-	-	-	-	-	-	-	-	-	-	-	-	-	-	-	-	-	-	-	-	-	-	-	±	-	-	++	-	-	-	-	-	-	++	-	-	-	++	++	++	-	-	-	++	-	-	-	-	-
Tooth	Inflammation	-	-	-	-	-	-	-	-	-	-	-	-	-	-	-	-	-	-	-	-	-	-	-	-	-	-	-	-	-	-	-	-	-	-	-	-	-	-	-	-	-	-	-	-	-	-	-	-	-
	Single cell death	-	-	-	-	-	-	-	-	-	-	-	-	-	-	-	-	-	-	-	-	-	-	-	±	-	-	++	-	-	-	-	-	-	-	-	-	-	-	-	-	-	-	-	-	-	-	-	-	-
Bone	Single cell death	-	-	-	-	-	-	-	-	-	-	-	-	-	-	-	-	-	-	-	-	-	-	-	-	-	-	-	-	-	-	-	-	-	-	-	-	-	-	-	++	-	-	-	++	-	-	-	-	-
Eyes	Ganglion cell loss	-	-	-	-	-	-	-	-	-	-	-	-	-	-	-	-	-	-	-	-	-	-	-	-	-	±	±	-	-	-	-	-	-	±	-	-	-	++	++	++	-	-	-	++	-	-	-	-	-
Olfactory bulb	Vacuolation	-	-	-	-	-	-	-	-	-	-	-	-	-	-	-	-	-	-	-	-	-	-	-	-	-	-	-	-	-	-	-	-	-	-	-	-	-	++	++	++	-	-	-	++	-	-	-	-	-
	Single cell death	-	-	-	-	-	-	-	-	-	-	-	-	-	-	-	-	-	-	-	-	-	-	-	-	-	-	-	-	-	-	-	-	-	-	-	-	-	++	++	++	-	-	-	++	-	-	-	-	-
Cerebrum	Inflammation	-	-	-	-	-	-	-	-	-	-	-	-	-	-	-	-	-	-	-	-	-	-	-	-	-	-	-	-	-	-	-	-	-	-	-	-	-	++	++	++	-	-	-	++	-	-	-	-	-
	Single cell death	-	-	-	-	-	-	-	-	-	-	-	-	-	-	-	-	-	-	-	-	-	-	-	-	-	-	-	-	-	-	-	-	-	-	-	-	-	4d	4d	4d	-	-	-	4d	-	-	-	-	-
Cerebellum	Inflammation	-	-	-	-	-	-	-	-	-	-	-	-	-	-	-	-	-	-	-	-	-	-	-	-	-	-	±	±	-	-	-	-	-	-	-	-	-	++	++	++	-	-	-	++	-	-	-	-	-
	Single cell death	-	-	-	-	-	-	-	-	-	-	-	-	-	-	-	-	-	-	-	-	-	-	-	-	-	-	-	-	-	-	-	-	-	-	-	-	-	-	-	-	-	-	-	-	-	-	-	-	-
Brain stem	Inflammation	-	-	-	-	-	-	-	-	-	-	-	-	-	-	-	-	-	-	-	-	-	-	-	-	-	-	±	-	-	-	-	-	-	-	-	-	-	±	±	±	-	-	-	±	-	-	-	-	-
	Single cell death	-	-	-	-	-	-	-	-	-	-	-	-	-	-	-	-	-	-	-	-	-	-	-	-	-	-	-	-	-	-	-	-	-	-	-	-	-	-	-	-	-	-	-	-	-	-	-	-	-
Spinal Cord	Vacuolation	-	-	-	-	-	-	-	-	-	-	-	-	-	-	-	-	-	-	-	-	-	-	-	-	-	-	±	-	-	-	-	-	-	-	-	-	-	±	±	±	-	-	-	±	-	-	-	-	-
	Single cell death	-	-	-	-	-	-	-	-	-	-	-	-	-	-	-	-	-	-	-	-	-	-	-	-	-	-	-	-	-	-	-	-	-	-	-	-	-	3mf	2mf	2mf	-	-	-	2mf	-	-	-	-	-
Lung	Hemorrhage	-	-	-	-	-	-	-	-	-	-	-	-	-	-	-	-	-	-	-	-	-	-	-	-	-	-	-	-	-	-	-	-	-	-	-	-	-	±	±	++	-	-	-	++	-	-	-	-	-
	Inflammation	-	-	-	-	-	-	-	-	-	-	-	-	-	-	3mf	2mf	2mf	3mf	2mf	3mf	2mf	4mf	3mf	4mf	-	3mf	2mf	2mf	2fe	2mf	2fe	-	2mf	2mf	2mf	-	3mf	-	-	2mf	3mf	3mf	4mf	2fe	2fe	-	3mf	3mf	3fe
	Single cell death	-	-	-	-	-	-	-	-	-	±	±	±	±	±	++	++	±	++	±	±	±	++	++	++	±	±	++	++	±	±	±	-	±	±	-	-	-	±	-	-	±	-	-	-	-	-	±	±	±
Repro tract	Single cell death	-	-	-	-	-	-	-	-	-	-	-	-	-	-	-	-	-	-	-	-	-	-	-	-	-	-	-	-	-	-	-	-	-	-	-	-	-	-	-	-	-	±	±	±	±	-	±	±	±

Symbols (++, ±, −) indicate whether the lesion was present and easily recognized (++); variably present throughout the tissue (±); or not detected histologically (−). A score of 1–5 indicates the severity of the inflammation: 1 (minimal); 2 (mild); 3 (moderate); 4 (marked); 5 (severe). Distribution of the lesion: f (focal); fe (focally extensive); mf (multifocal); d (diffuse)

**Fig. 4:**
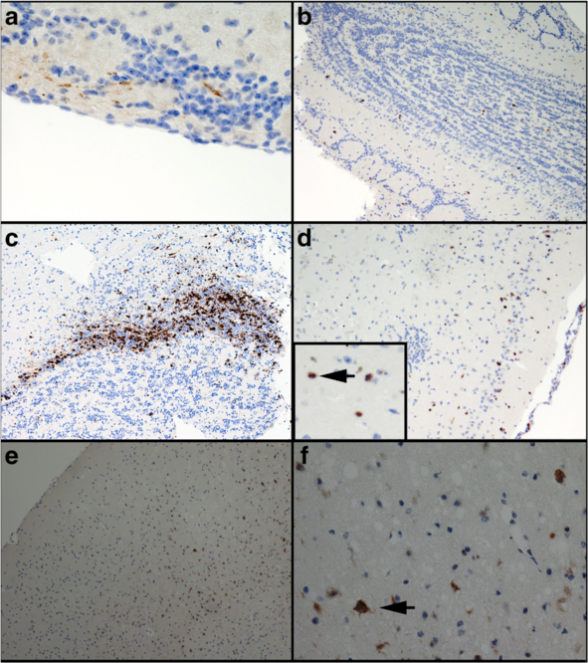
Immunohistochemical findings for cleaved-caspase 3 (**a**-**d**) and LC3BII (**e**-**f**) in mice after AE infection with EEEV strain FL93-939. Mice were infected with approximately 100LD_50_ and were euthanized at specified time points (n = 5). While cleaved-caspase 3 antigen was detected in very few cells in the olfactory bulb (**a** magnification X400), there was increased antigen detected in the olfactory bulb by 2 dpi (**b** magnification X100). The increase in cleaved-caspase 3 antigen detection in the olfactory bulb continued through the study and was present in clusters of cells at 4 dpi (**c** magnification X100). Cleaved-caspase 3 antigen was present within the cerebrum, primarily within the piriform cortex (**d** magnification X100) and specifically within cells with karyorrhexis (**d** inset, arrow) as well as within some inflammatory cells within the meninges. The LC3BII antigen was detected in low numbers of neurons within the olfactory bulb, frontal cortex (**e** magnification X100), and midbrain at 3–4 dpi. The antigen was cytoplasmic and punctate in affected cells (**f** magnification X400, arrow)

In an attempt to further elucidate the mechanism of neuronal cell death, brain sections from the AE study were evaluated immunohistochemically for the presence of cleaved-caspase-3 antigen as a marker of apoptosis as well as LC3BII antigen as a marker of autophagy. While there were very few cells in the control animals that had intracytoplasmic staining for cleaved-caspase-3 antigen (Fig. 4a), there was a marked increase in the number of cells staining for the apoptotic marker in the olfactory bulb of EEEV infected mice, especially after 2 dpi (Fig. 4b), this trend continued through 4 dpi (Fig. 4c). The cleaved-caspase 3 antigen was also detected within the cerebrum, especially within the piriform cortex (Fig. 4d) and within cells with karyorrhexis (Fig. 4d, inset, arrow) as well as within some inflammatory cells within the meninges. The antigen remained present within the cerebrum, especially the piriform cortex, at low levels through 4 dpi, the study endpoint.

LC3BII is typically noted as cytoplasmic punctate staining in cells undergoing autophagy. While autophagy can be part of normal cellular turnover in the brain, staining was not evident in uninfected control animals. Overall, there were only a few neurons containing intracytoplasmic punctate staining; however, these were in areas that were positive for viral antigen. LC3BII antigen was noted in 3/5 mice in the olfactory bulb, in both the external plexiform and internal granular layers, at 3 dpi. At 4 dpi, 6 out of 10 animals had low numbers of neurons in the olfactory bulb, frontal cortex (Fig. 4e), and midbrain which contained intracytoplasmic punctate staining (Fig. 4f, arrow) for LC3BII. This suggests that while autophagy occurs in EEEV infected mice, it does not occur until later in the disease and that only a small number of neurons are removed by this process.

## Discussion

In order to better understand the pathogenesis of EEEV strain FL93-939 several studies were conducted to evaluate the differences between three routes of infection: subcutaneous, aerosol, and intranasal. Subcutaneous inoculation is intended to mimic a mosquito-acquired infection. Aerosol exposure is a likely route in the event of an intentional release of EEEV and accidental laboratory exposure. Finally, intranasal exposure is often used by labs as a substitute for aerosol infection by labs not possessing the specialize equipment necessary to carry out an aerosol exposure, necessitating a comparison of the two routes. Although the intranasal and aerosol routes had similar findings, there were important pathological differences noted, most prominently that viral antigen was detected in the brain at 1 dpi in the AE study as compared to 2 dpi in the IN study; that viral antigen was present in the brain of most animals in the AE study by 2 dpi and throughout the brain by 3 dpi. This increasingly rapid spread in the AE study correlated with clinical signs and animals met study end points 1 day earlier than those in the IN study.

In additional studies, we used virus titration of tissue homogenates to complement the histopathology and immunohistochemistry presented here and further define the pathogenesis of EEEV strain FL93-939 in mice [10]. Not surprisingly, viral titration was much more sensitive than IHC in detecting the presence of virus at early time points. In the IN study, virus was first detected in the blood and the brain homogenate at 1 dpi; however, at this time, viral antigen was only noted in the nasal cavity (olfactory epithelium and lamina propria) and not the olfactory bulb or cerebrum immunohistochemically. The bipolar olfactory neuron, found in the olfactory epithelium is instrumental as one pole of the cell has cilia that project into the air passages, a likely site of viral contact, and the opposite pole extends an axon that synapses directly with neurons of the olfactory bulb. Therefore, viruses that target olfactory neurons have a direct conduit to the brain. This appeared to be the case in this study, because by 2 dpi, when viremia peaked and titer in the brain rapidly increased, there was viral antigen present multifocally within the nasal cavity and also within the olfactory bulb. In the one animal in which viral antigen was detected in the frontal cortex and midbrain, it was present in only a few scattered cells primarily within the piriform cortex. Virus then appeared to spread in a rostral to caudal fashion, infecting neurons of the cerebellum and brain stem only at the later time points. Overall, these findings suggest the virus entered the brain via the olfactory route rather than the vascular route, which is similar to that seen in guinea pigs exposed to aerosolized EEEV [11].

The results for the AE study were very similar to the results of the IN study, with the important exception that virus was detected in the brain by titer at only 6 hpi and viral antigen was present in the olfactory bulb by 1 dpi, a day earlier than in the IN study. The virus appeared to enter the brain through the olfactory tract and spread transneuronally, rostral to caudal, as noted in the IN study; however this occurred more rapidly and more frequently at each time point. The aerosol delivery method likely allowed more viral contact with olfactory neurons, thus facilitating the earlier viral invasion of the olfactory bulb and subsequent transport to the olfactory tract and beyond. These results support the clinical findings of more rapid and severe disease onset in this study compared to the IN or SC studies.

The results of the SC study were less clear, which may be due to the dose and/or route of inoculation. Throughout the study, evidence of viral infection was not observed consistently. While virus was first detected in the brain by standard plaque assay at 1 dpi (1 of 5 animals, 20 %), virus was only detected in 10 of 60 (17 %) of animals from 3–8 dpi. Similar inconsistencies were observed on pathological examination of the tissues. Viral antigen was only detected in the nasal cavity and/or brain in 10 of 60 animals from 3–8 dpi. In some animals, viral antigen was detected in the nasal cavity and olfactory bulb, while in others it was detected in the nasal cavity and throughout the brain simultaneously. This could be a result of individual animal variability or the result of vascular spread to the brain. These results are similar to those found by Vogel et al. [12]; however, in that study it was noted that the virus generally spared the olfactory epithelium. Similar to their study, viral replication was observed at the inoculation site, in fibroblasts, skeletal myocytes, and synovial cells; however, unlike in the Vogel study, no viral antigen was detected in osteoblasts of the long bones in this study. This is likely due to the age difference of the mice used in each of the studies. In the Vogel study, the mice were 5-weeks old and the mice were actively growing with open growth plates and high numbers of osteoblasts, whereas in the current study the mice were 12-weeks-old, which is considered an adult characterized by closed growth plates and relatively few osteoblasts. Also, in contrast to the Vogel study, a few animals in this SC study had moderate to marked meningoencephalitis with vasculitis, thrombosis, and hemorrhage at 6–7 dpi. Interestingly, viral antigen was not present adjacent to affected vessels, suggesting this lesion could be immune-mediated rather than a direct result of viral infection. Meningoencephalitis and vasculitis have been associated with EEEV after natural infection in humans and aerosol exposure in guinea pigs [11], but have not been previously reported in mice. Our findings may be due to the lower inoculation dose and/or the prolonged study design, which may actually mimic natural human infection more accurately.

In humans, the incubation period following natural infection is short, usually 4–10 days; however, in most cases the exact time of exposure is not known. Systemic infection is often characterized by abrupt onset of chills and fever followed by malaise, arthralgia, and myalgia. Typically, these are difficult parameters to measure in animals; however, telemetry did allow monitoring of temperature and activity and fever and decreased activity (lethargy or malaise) were observed in many infected animals. Clinical signs of encephalitis in humans include abrupt onset of fever, intense headache, irritability, restlessness, drowsiness, anorexia, nausea, vomiting, diarrhea, cyanosis, convulsions, and coma. Again, while most of these clinical signs cannot be evaluated in mice, marked lethargy and tremors were noted in some infected animals. In humans, death usually occurs within 2–14 days after the onset of clinical signs [1]. Similarly, mice in these studies generally were moribund or succumbed to infection within 2–4 days following the onset of clinical signs. The slightly longer time course in humans may be due to species differences or the more intensive supportive care typically provided to humans with neuroinvasive disease. Histopathologic lesions in human cases of EEE include vasculitis, thrombosis, perivascular cuffing, neutrophilic and histiocytic infiltrates, neuronal cell death, neuronophagia, focal necrosis, demyelination, and gliosis [13]. While vasculitis, thrombosis, and inflammation were not prominent lesions in the IN or AE studies, these histopathologic changes were noted in several animals, especially after 5 dpi, in our SC study. Neuronal cell death, regardless of mechanism of cell death, is a universal key feature in this disease and has been noted in all animal models studied [6, 11, 12, 14]. Again, the lower dose, slightly longer clinical course, and the histopathological lesions noted in the SC study likely more closely mimic the natural disease seen in humans, making this mouse model a useful tool for further investigation and evaluation of medical countermeasures. Additionally, the results presented here support the finding by Roy et. al. in which aerosolized EEEV in guinea pigs entered the brain through the olfactory system followed by transneuronal spread to all regions of the brain with viral antigen detected immunohistochemically in the olfactory mucosa, the olfactory nerves, and/or lamina propria 1 dpi [11]. To date, no published NHP studies have evaluated the mechanism of neuroinvasion of EEEV following aerosol exposure [6, 14–16]. While it is likely that EEEV utilizes the olfactory system to invade the brain in NHP, this is an important question that remains to be answered.

To further elucidate the mechanism of neuronal cell death in the mouse model, brain sections from the AE study were evaluated for the presence of cleaved-caspase 3 antigen as a marker of apoptosis as well as LC3B-II antigen as a marker of autophagy. While there was a low basal level of apoptosis present in control animals, significantly more was present in EEEV infected animals, especially at later time points. Cleaved-caspase 3 antigen was found primarily in the neurons of the olfactory bulb and the piriform cortex of the cerebrum. However, the number of cells staining for cleaved-caspase-3 was lower than expected based on histologic findings. At later time points, a significant number of neurons with condensed cytoplasm and either pyknosis or karyorrhexis, suggestive of apoptosis, were present. This disparity may be due to the fact that cleaved-caspase 3, a marker in the terminal pathway of apoptosis, may not be present in cells that have already undergone apoptosis with resultant cellular and nuclear fragmentation. On the other hand, it may be that some neurons are not undergoing apoptosis but are dying by another mechanism, such as necrosis or autophagy. Autophagy, also known as type II programmed cell death, has been recognized as a means of cellular death in other alphaviral infections [17, 18] and can be histologically indistinguishable from apoptosis. We found relatively few neurons that were immunohistochemically positive for LC3B-II antigen, and these were only present at 3–4 dpi. The positive neurons were within viral antigen positive areas within the olfactory bulb, frontal cortex, and midbrain, but specifically not within the piriform cortex. This suggests that neurons in various areas of the brain may respond to viral infection differently and ultimately undergo one of several mechanisms of cell death.

It is important to note that histologically it can be difficult to determine the mechanism of cell death for each individual neuron as apoptosis, necrosis, and autophagy can occur simultaneously in the same tissue. The morphological features of apoptosis are generally characterized by cell shrinkage and convolution, pyknosis and karyorrhexis, intact cell membranes with no inflammation affecting single cells or small clusters of cells, while the morphological features of necrosis are generally characterized by cell swelling, karyolysis, and disruption of cell membranes with inflammation affecting contiguous cells. However, pyknosis and karyorrhexis can be seen in necrosis as well as apoptosis [19] and recent studies indicate that necrosis may not only be an accidental form of cell death, but that it may be initiated or modulated by programmed control mechanisms, much like apoptosis and autophagy [20–23]. There is overlap between these two processes, which has been described as the “necrosis-apoptosis continuum” and whether a cell dies by necrosis or apoptosis may be determined by the tissue type, developmental stage of the tissue, cell death signal, as well as the physiologic microenvironment [24, 25]. Autophagy is a more recently recognized form of programmed cell death. While autophagy is generally recognized as an adaptive response, there is some controversy as to its role in cell death. It is uncertain if the accumulation of autophagosomes in some dying cells is a consequence of cellular adaptation alone or if these structures actually facilitate cell death [22]. As the molecular pathways of these processes become more defined, it may be revealed that the various mechanisms of cell death work in concert to eliminate unwanted cells in order to preserve tissue and organ function.

It is widely accepted that the brain is composed of numerous morphologically, metabolically, and functionally diverse neuroanatomic regions, which have differential sensitivities to various toxic and infectious insults. While neurons can be broadly classified as “small neurons” and “large neurons”, various subtypes of each exist and interact with a number of support cells of varying function [26]. With the complexity and mutually supportive roles of the numerous cell types within the CNS just beginning to be understood, it is not difficult to imagine that one or more mechanisms of cell death may play an important role in overall maintenance of brain function. As our study suggests, in mice infected with EEEV, neurons in the CNS may undergo cell death by apoptosis, necrosis, or autophagy depending on the neuroanatomic location of the neuron and stage of disease. Ultimately, if one or more mechanisms of cell death are consistently identified in animal models of EEEV, these could represent additional targets for therapeutic intervention. If neuronal death could be prevented or if fewer neurons are lost during neuroinvasive disease, this could significantly improve the long term neurological sequelae that are often noted in human cases.

## Conclusion

While it has long been known that in mice infected with VEEV, regardless of route of exposure, neuroinvasion occurs through the olfactory system [6, 27–30], this study has been crucial in understanding the mechanism of neuroinvasion of EEEV. It is clear from these studies that EEEV enters the brain through the olfactory system when mice are exposed either by the intranasal or aerosol route, with the aerosol route resulting in neuroinvasion approximately 1 day earlier than the nasal route. The mechanism and rapidity of virus entry into the brain has important vaccine and therapeutic implications. First, for a vaccine to be effective, it must prevent the virus from infecting olfactory neurons. Since the nasal cavity is a mucosal surface, it would be reasonable to expect that an effective vaccine would induce the production of neutralizing IgA as well as IgG. Secondly, since viral antigen was present within the brain at 1 dpi in the aerosol study, a useful therapeutic would need to be administered very soon after exposure and would have to be formulated to easily cross the blood–brain-barrier. Lastly, while the subcutaneous route most closely mimics human disease, when developing medical countermeasures, it is important to test them against multiple routes of infection, including aerosol or intranasal. These are not insurmountable tasks; however, these factors must be taken into account in the development of vaccines, post-exposure prophylaxis and therapeutics for EEEV infection.

## Methods

### Mice

Specific pathogen free, 8–10 week-old female BALB/c mice (NCI, Frederick, MD) were housed in cages equipped with microisolators and were provided food and water *ad libitum* throughout the study. The room temperature was 23 ± 1 °C and periods of light and dark were maintained on a 12 h cycle. Mice were acclimated for 1 week then housed in a biosafety level 3 (BSL-3) facility. Research was conducted under an IACUC approved protocol in compliance with the Animal Welfare Act, Public Health Service Policy, and other Federal statutes and regulations relating to animals and experiments involving animals. The facility where the research was conducted is accredited by the Association for the Assessment and Accreditation of Laboratory Animal Care International and adheres to principles stated in the Guide for the Care and Use of Laboratory Animals, National Research Council, 2011.

### Virus

EEEV strain FL93-939 was obtained from Dr. Scott Weaver, UTMB, Galveston, TX. A sucrose-purified working stock was prepared from seed stock (P1) through an additional passage (P2) in Vero cells. Virus titer was determined by standard plaque assay on Vero cell monolayers. Challenge virus was diluted in either Eagle’s minimum essential medium (EMEM) (Cellgro, Mediatech, Manassas, VA) or sterilized phosphate buffered saline (PBS) (GIBCO Invitrogen Corp., Grand Island, NY).

### Experimental design

Groups of 10 mice were exposed to approximately 30-100LD_50_ of EEEV strain FL93-939 by either the intranasal, aerosol or subcutaneous route. For the intranasal route of exposure, virus dose was prepared in a 20 μL volume in sterilized PBS. Control mice received only sterilized PBS. Mice were briefly anesthetized with isoflurane using the IMPAC^6^ (VetEquip, Inc., Pleasanton, CA) and given 10 μL of challenge virus per nostril. For the aerosol route of exposure, virus dose was prepared in a 10 ml volume in EMEM. Control mice were exposed to diluent only. Aerosol exposures were conducted in a whole-body bioaerosol exposure system. A Collison nebulizer (BGI, Inc., Waltham, MA) was used to generate small (1 μm mass median aerodynamic diameter) diameter particles for each acute 10 min exposure. Briefly, mice were placed in wire cages, which were then placed into a chamber where they were exposed to aerosolized virus for 10 min. ‘Presented’ dose was estimated by calculating the respiratory minute volume (V_m_) using Guyton’s formula [31], expressed as Vm = 2.10 x W_b_^0.75^ where W_b_ = body weight (gm) based on the average group weights the day of exposure. The presented dose was then calculated by multiplying the estimated total volume (V_t_) of experimental atmosphere inhaled by each animal (V_t_ = V_m_ x length of exposure) by the empirically determined exposure concentration (C_e_) (‘presented dose’ = C_e_ x V_t_). Exposure concentration, expressed in plaque-forming units (PFU)/L, was determined by isokinetic sampling of the chamber with an all-glass impinger (AGI) (Ace Glass, Vineland, NJ). Samples were titrated by standard plaque assay on Vero cell monolayers [32]. For the subcutaneous route of exposure, virus dose was prepared in a 10 μL volume in EMEM. Mice were inoculated in the left foot pad in order to track viral replication in the surrounding tissue and draining lymph node (popliteal lymph node). Control mice received diluent only. Challenge virus preparations were back-titrated by standard plaque assay using Vero cells. Mice from the intranasal and aerosol studies were euthanized at pre-determined time points: 6, 12, 24, 48, 72, 96, and 120 hours post-infection (hpi). In addition to the previous listed time points, mice in the subcutaneous study were also euthanized at 144, 168, and 192 hpi. At the time of euthanasia, mice were anesthetized with mouse K-A-X (50 mg ketamine (Fort Dodge Animal Health, Fort Dodge, IA), 0.5 mg acepromazine (Boehringer Ingelheim, Ridgefield, CT), and 5.5 mg xylazine (Lloyd Laboratories, Walnut, CA) given intraperitoneal at a dose of 0.2 ml per 20 gm. There were 10 mice total for each time point, five mice from each time point were euthanized and perfused with saline and tissues were harvested for virus titer analysis and the other five mice from each time point were euthanized and perfused with 10 % neutral buffered formalin (NBF) (LabChem, Inc., Pittsburg, PA) and tissues were harvested for histopathologic analysis.

### Pathology

Animal tissues collected for pathology were fixed in 10 % NBF for a minimum of 21 days prior to removal from the BSL-3 containment lab for processing. Skulls were decalcified in 10 % EDTA in Tris buffer solution (pH 6.95) (Sigma Chemical Co., St. Louis, MO). Tissues were trimmed and processed according to standard protocol and embedded in paraffin blocks. Histologic sections were trimmed at 5–6 μm thickness, mounted on positively charged glass slides (Superfrost Plus, Fischer Scientific, Pittsburg, PA), and stained with hematoxylin and eosin (PolyScientific, Bay Shore, NY).

Serial sections were stained for viral antigen using a polyclonal rabbit antiserum directed against several alphaviruses, followed by a horseradish peroxidase-labeled polymer conjugated to goat anti-rabbit immunoglobulins. Briefly, tissue sections were deparaffinized using Xyless (LabChem, Inc.) and rehydrated using sequentially less concentrated alcohol solutions ranging from 100 % to 70 %. Endogenous peroxidases were blocked using a methanol/hydrogen peroxide solution. To increase staining intensity, antigen retrieval was performed by immersing slides in Tris/EDTA buffer for 30 min at 97 °C. Endogenous proteins were blocked by incubating the slides in serum-free protein block (Invitrogen, Carlsbad, CA) plus 5 % normal goat serum (Vector Labs, Burlingame, CA) for 30 min at room temperature. Sections were incubated with the primary antibody, a polyclonal rabbit antiserum directed against EEEV, WEEV, VEEV and Sindbis virus (Applied Diagnostic Branch, Diagnostic Systems Division, USAMRIID) diluted 1:8000, for 30 min at room temperature. Sections were then incubated with a secondary antibody, horseradish peroxidase-labeled polymer conjugated to goat anti-rabbit immunoglobulins (DAKO, Carpenteria, CA), and incubated for 30 min at room temperature. Staining was completed by adding the substrate-chromagen, diaminobenzidine (DAB) (DAKO) and incubating slides for 5 min at room temperature. Tissues were counterstained with hematoxylin for 2 min at room temperature and then dehydrated in sequentially more concentrated alcohol solutions, cleared using Xyless II, and coverslip was mounted using Permount (Fisher Scientific). Non-immune (normal) rabbit serum (Vector Labs) was used as a negative control for the primary antibody. Sections of confirmed EEE virus-infected mouse brain were used as a positive control.

Serial sections were stained for cleaved caspase-3, a marker for apoptosis, using a monoclonal rabbit antiserum, followed by a horseradish peroxidase-labeled polymer conjugated to goat anti-rabbit immunoglobulins. Briefly, tissue sections were processed as described above. Sections were then incubated with the primary antibody, a monoclonal rabbit antiserum directed against cleaved caspase-3 (Asp175) (51AE) (Cell Signaling Technology, Danvers, MA) diluted 1:100, for 60 min at room temperature. Sections were then incubated with a secondary antibody, horseradish peroxidase-labeled polymer conjugated to goat anti-rabbit immunoglobulins (DAKO), and incubated for 30 min at room temperature. Staining was completed by adding the substrate-chromagen, diaminobenzidine (DAB) (DAKO) and incubating slides for 5 min at room temperature. Tissues were counterstained with hematoxylin for 2 min at room temperature and then dehydrated in sequentially more concentrated alcohol solutions, cleared using Xyless II, and coverslip was mounted using Permount. Non-immune (normal) rabbit serum (Vector Labs) was used as a negative control for the primary antibody. Sections of confirmed VEE virus-infected mouse spleen were used as a positive control.

Serial sections were also stained for LC3B II, a marker of autophagy, using a monoclonal rabbit antiserum, followed by a horseradish peroxidase-labeled polymer conjugated to goat anti-rabbit immunoglobulins. Briefly, tissue sections were processed as described above. Sections were then incubated with the primary antibody, a monoclonal rabbit antiserum directed against LC3B (DII) x P (R) (Cell Signaling) diluted 1:500, for 60 min at room temperature. Sections were then incubated with a secondary antibody, horseradish peroxidase-labeled polymer conjugated to goat anti-rabbit immunoglobulins (DAKO), and incubated for 30 min at room temperature. Staining was completed by adding the substrate-chromagen, diaminobenzidine (DAB) (DAKO) and incubating slides for 5 min at room temperature. Tissues were counterstained with hematoxylin for 2 min at room temperature and then dehydrated in sequentially more concentrated alcohol solutions, cleared using Xyless II, and coverslip was mounted using Permount. Non-immune (normal) rabbit serum (Vector Labs) was used as a negative control for the primary antibody.
